# A Combined Treatment with Berberine and Andrographis Exhibits Enhanced Anti-Cancer Activity through Suppression of DNA Replication in Colorectal Cancer

**DOI:** 10.3390/ph15030262

**Published:** 2022-02-22

**Authors:** Yinghui Zhao, Souvick Roy, Chuanxin Wang, Ajay Goel

**Affiliations:** 1 Department of Molecular Diagnostics and Experimental Therapeutics, Beckman Research Institute of City of Hope, Monrovia, CA 91010, USA; zyhjude@126.com (Y.Z.); soroy@coh.org (S.R.); 2 Department of Clinical Laboratory, The Second Hospital, Cheeloo College of Medicine, Shandong University, Jinan 250033, China; wcx6601@126.com; 3 Shandong Engineering & Technology Research Center for Tumor Marker Detection, Jinan 250033, China; 4 Shandong Provincial Clinical Medicine Research Center for Clinical Laboratory, Jinan 250033, China; 5 City of Hope Comprehensive Cancer Center, Duarte, CA 91010, USA

**Keywords:** colorectal cancer, berberine, Andrographis, DNA replication

## Abstract

The high morbidity and mortality associated with colorectal cancer (CRC) are largely due to the invariable development of chemoresistance to classic chemotherapies, as well as intolerance to their significant toxicity. Many pharmaceutical formulations screened from natural plant extracts offer safe, inexpensive, and multi-target therapeutic options. In this study, we demonstrated that *Berberis vulgaris* L. (Berberine) and *Andrographis paniculata* (Burm. f.) Nees (Andrographis) extracts exerted their synergistic amplified anti-cancer effects by jointly inhibiting cell viability, suppressing colony formation, and inducing cell cycle arrest. Consistent with our in-vitro findings, the amplified synergistic anti-cancer effects were also observed in subcutaneous xenograft preclinical animal models, as well as patient-derived primary tumor organoids. To explore the molecular mechanisms underlying the amplified synergistic anti-cancer effects, RNA sequencing was performed to identify candidate pathways and genes. A transcriptome analysis revealed that DNA-replication-related genes, including FEN1, MCM7, PRIM1, MCM5, POLA1, MCM4, and PCNA, may be responsible for the enhanced anticancer effects of these two natural extracts. Taken together, our data revealed the powerful enhanced synergistic anti-CRC effects of berberine and Andrographis and provide evidence for the combinational targeting of DNA-replication-related genes as a promising new strategy for the therapeutic option in the management of CRC patients.

## 1. Introduction

Colorectal cancer (CRC) ranks as the third most common malignancy worldwide and remains one of the leading causes of cancer-related deaths globally [1]. In 2021, an estimated 149,500 adults in the United States will be diagnosed with CRC, and approximately 53,000 will succumb to CRC-related deaths [2]. The survival benefits of patients with early-stage CRC have increased significantly over the past few decades, primarily due to the advances in early detection and the increased uptake of screening [3,4]. Nonetheless, the overall clinical outcomes in patients with CRC have been unsatisfactory, especially in patients with advanced disease [5]. Surgical resection for the complete removal of the tumor is the only curative treatment for localized CRC but is no longer effective in advanced cases with metastatic lesions, which account for ~25% of all diagnosed cases [6,7]. Given the higher disease incidence and diagnosis of cancer at advanced stages, chemotherapy, rather than surgery, has become the standard approach for the management of such patients [8]. However, the therapeutic benefit of chemotherapy has been limited due to the invariable development of drug resistance, as well as the toxicity from these drugs, that a large majority of patients experience [9]. Thus, the identification of more effective and safe compounds that can be used as rational treatment choices with greater therapeutic outcomes is urgently needed.

Although there is currently no effective way to overcome de novo or acquired chemoresistance to classic chemotherapeutic drugs, data in recent years has provided clear evidence that the application of combined regimens, encompassing multiple agents that simultaneously target distinct cancer growth signaling pathways, may be a more attractive therapeutic option and more effectively prolong survival in patients with CRC [10]. From the perspective of clinical needs, such multiagent combinations, rather than sequential treatments with individual drugs, tend to result in lower doses and exhibit fewer harmful side effects and less dose-limiting toxicity [11,12]. Natural products derived from plants have always been an important source for developing novel pharmacologically active drugs [13]. Consequently, many anti-cancer drugs that originated from natural-product-derived compounds are currently used in the clinic to treat patients with CRC [14]. In this context, it is necessary to develop more effective and safe drug combination regimens that combine low-dose natural compounds to reduce toxicity.

Berberine, a major isoquinoline alkaloid constituent of many medicinal herbs, such as *Coptidis rhizome* and *Berberis vulgaris*, has shown great promise as an anti-cancer agent [15]. Studies have reported several important molecular mechanisms responsible for the anti-cancer effect of berberine. This natural medicine can inhibit the growth of intestinal polyps in patients with familial adenomatous polyposis by downregulating c-Myc [16]. In addition, berberine inhibits tumorigenesis associated with colitis via inhibition of EGFR-signaling-related cancer cell growth [17]. In a similar context, andrographolide is one of the principle diterpenoid lactones isolated from herb *Andrographis paniculata* [18].Studies from our group and others have confirmed the anti-tumorigenic properties of Andrographis, which is a potent phytoconstituent present in *Andrographis paniculate* extracts, by inhibiting CRC cell growth through the activation of ferroptosis and synergistically enhancing the therapeutic efficacy of conventional chemotherapeutic drugs to reduce their side effects [19,20,21]. Considering that berberine and Andrographis demonstrate their anti-tumor properties through distinct pathways and given the consensus that more effective anti-cancer therapies tend to be multi-targeted, we hypothesized that a combination therapy with these two naturally occurring compounds might offer an enhanced anti-tumorigenic activity in CRC. In this study, we sought to interrogate whether the combination of berberine and Andrographis enhances the anti-cancer potential in CRC and the molecular mechanisms underlying the enhanced anti-cancer effect.

To interrogate this hypothesis, systematic and comprehensive approaches were performed in the present study, which involved cell culture and an animal xenograft model, as well as patient-derived tumor organoid experiments. We, for the first time, revealed that Andrographis enhanced the anti-cancer activity of berberine in CRC. Our data revealed that the combination of these two extracts exhibited a more significant cancer inhibitory effect than individual treatment in CRC cells, xenograft models, as well as in patient-derived tumor organoids. Genome-wide expression profiling of CRC cells demonstrated that berberine and Andrographis combine to cooperatively inhibit CRC by regulating DNA replication. In summary, this study proposed the enhanced anticancer effects of berberine and Andrographis and preliminarily explored the underlying mechanism, highlighting the potential application of the above two extracts in a therapeutic role for patients with CRC.

## 2. Results

### 2.1. A Combined Berberine and Andrographis Treatment Demonstrates Enhanced Activity to Suppress CRC Cell Growth

**Figure 1:**
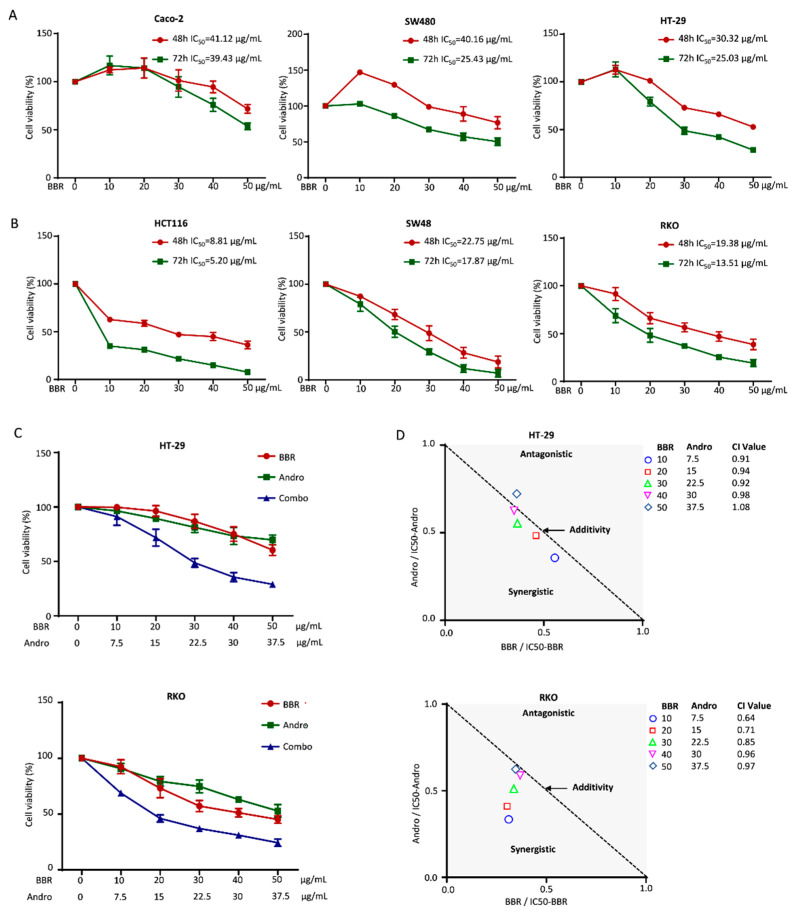
The combination of berberine and Andrographis interactively inhibited the growth of CRC cells. (**A**) Results of the CCK-8 assay showing the viability inhibition of MSI-type CRC cells following exposure to different concentrations of berberine for 48 h or 72 h; (**B**) The cell viability of MSS-type CRC cells treated with different concentrations of berberine for 48 h or 72 h; (**C**) Results of the CCK-8 assay to compare cell viability following treatment with berberine, Andrographis, and their combination for 48 h in the HT-29 and RKO cell lines; (**D**) Isobologram analysis of the CI values based on the results of the CCK-8 assay to determine the synergistic effects of berberine and Andrographis in the HT-29 and RKO cell lines. BBR, berberine; Andro, Andrographis; Combo, combination; CI, combination index.

To investigate the role of berberine in the growth inhibition of CRC cells, we first examined cell viability in a panel of human CRC cell lines in the presence of berberine. The human CRC lines broadly represent the common mutations and microsatellite status, including microsatellite stability (MSS) and microsatellite instability (MSI) types. The MSS (Caco-2, SW480, and HT-29) and MSI cell lines (HCT116, SW48, and RKO) were treated with increasing doses of berberine (0 to 50 μg/mL) for 48 or 72 h, respectively, and cell viability was detected by the CCK-8 assay. The results of the CCK-8 assays showed that berberine inhibited cell viability in a dose- and time-dependent manner. The IC_50_ values of Caco-2, SW480, HT-29, HCT116, SW48, and RKO were, respectively, 41.12 μg/mL, 40.16 μg/mL, 30.32 μg/mL, 8.81 μg/mL, 22.75 μg/mL, and 19.38 μg/mL after 48 h treatment (Figure 1A), and were 39.43 μg/mL, 25.43 μg/mL, 25.03 μg/mL, 5.20 μg/mL, 17.87 μg/mL, and 13.51 μg/mL after 72 h treatment (Figure 1B). Our results indicated that berberine generally had a stronger anti-cancer effect in MSI-type CRC cell lines.

The anti-tumorigenic properties of Andrographis in CRC have been well-established in our previous study and are more evident when used in combination with other anti-carcinogens [19]. To investigate the potential interactive effect of Andrographis and berberine on CRC cell growth, we conducted a drug combination study based on the Chou–Talalay method in the RKO and HT-29 cell lines, which represent the MSI and MSS CRC cell types. The results indicated that each extract individually could inhibit the growth of RKO and HT-29 cells, but their combination showed the strongest antiproliferative effects (Figure 1C). The final calculated combination index (CI) values of the berberine–Andrographis combinations were less than 1 in both cell lines (Figure 1D). A CI value < 1 indicates drug synergism, suggesting the synergistic anti-cancer effect of Andrographis and berberine in RKO and HT-29 cells.

### 2.2. Andrographis in Combination with Berberine Induces Cell Cycle Arrest and Inhibition of Cell Proliferation

**Figure 2:**
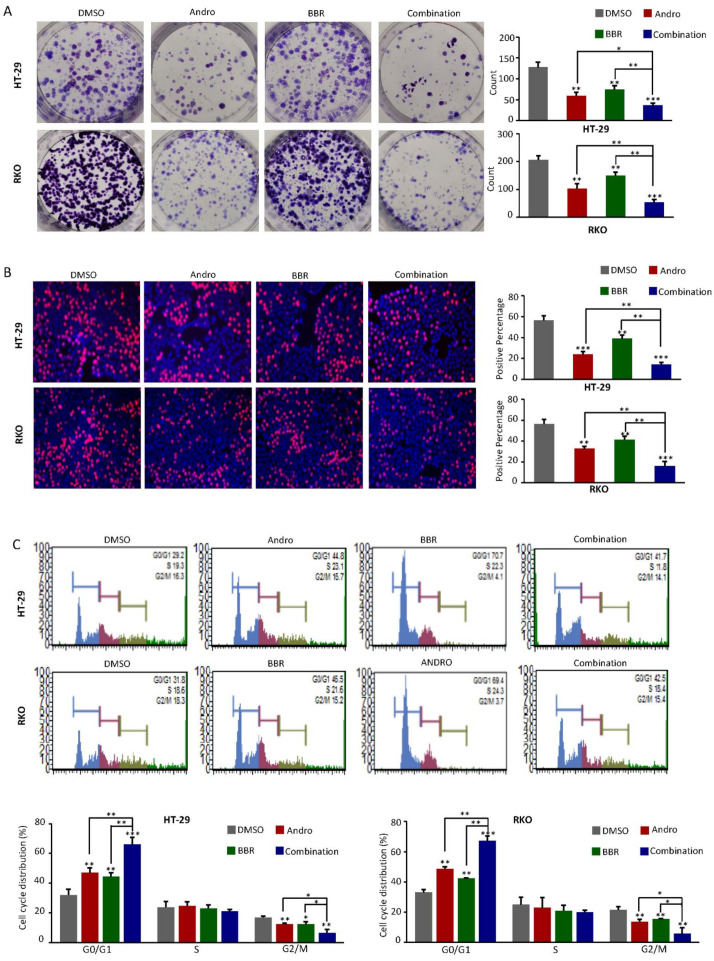
Combined Andrographis- and berberine-induced cell cycle arrest and inhibited cell proliferation. (**A**) Representative results of the colony formation assay in HT-29 and RKO cells after exposure to Andrographis and berberine alone and in combination for 7 days. The colony count analysis is shown in the right panel; (**B**) Representative results of the EdU assay (EdU, red; Hoechst, blue) after exposure to Andrographis and berberine alone and combination for 48 h. The quantitative analysis of the EdU-positive cells is shown in the right panel; (**C**) Representative histograms for the cell cycle distribution in HT-29 and RKO cells after treatment with Andrographis and berberine alone and in combination for 48 h. Values are expressed as the percentage of cells in the G0/G1, S, and G2/M phases of the cell cycle in the lower panel. Statistical Significance: * *p* < 0.05, ** *p* < 0.01, *** *p* < 0.001. BBR, berberine; Andro, Andrographis.

In order to more intuitively reflect the above synergistic inhibitory effect of CRC, we next investigated the combinational anti-cancer effects of berberine and Andrographis using the colony formation assay and EdU staining assays. Compared with the corresponding control groups, the clonal formation capacities of the CRC cells were drastically inhibited (*p* < 0.05), with significant cell proliferation inhibition (*p* < 0.05) after berberine or Andrographis treatment alone, in both the RKO and HT-29 cell lines. Notably, the combination of berberine and Andrographis treatment showed a more marked reduction in colony formation capacity and cell proliferation than treatment with either extract alone (*p* < 0.01; Figure 2A,B).

The roles of berberine and Andrographis on the cell cycle phase distribution were assessed by flow cytometry with propidium iodide as a probe. The cell cycle analysis revealed that both berberine and Andrographis could induce the percentage of cells in the G0/G1 phase to significantly increase (*p* < 0.01), with a concurrent decrease in the G2/M-phase cell fraction (*p* < 0.05) in the RKO and HT-29 cell lines, further confirming the growth inhibitory properties of both natural extracts. Consistent with the results of the colony formation and EdU assays, the combined treatment with berberine and Andrographis cooperatively induced cell cycle arrest in CRC cells (*p* < 0.05; Figure 2C). Taken together, these data indicate superior anti-cancer properties of the combination of berberine and Andrographis over each agent alone, by inhibiting cell proliferation, suppressing colony formation capacity, and inducing cell cycle arrest.

### 2.3. Andrographis Enhances the Efficacy of Berberine in Mouse Xenografts and Patient-Derived Tumor Organoids

**Figure 3:**
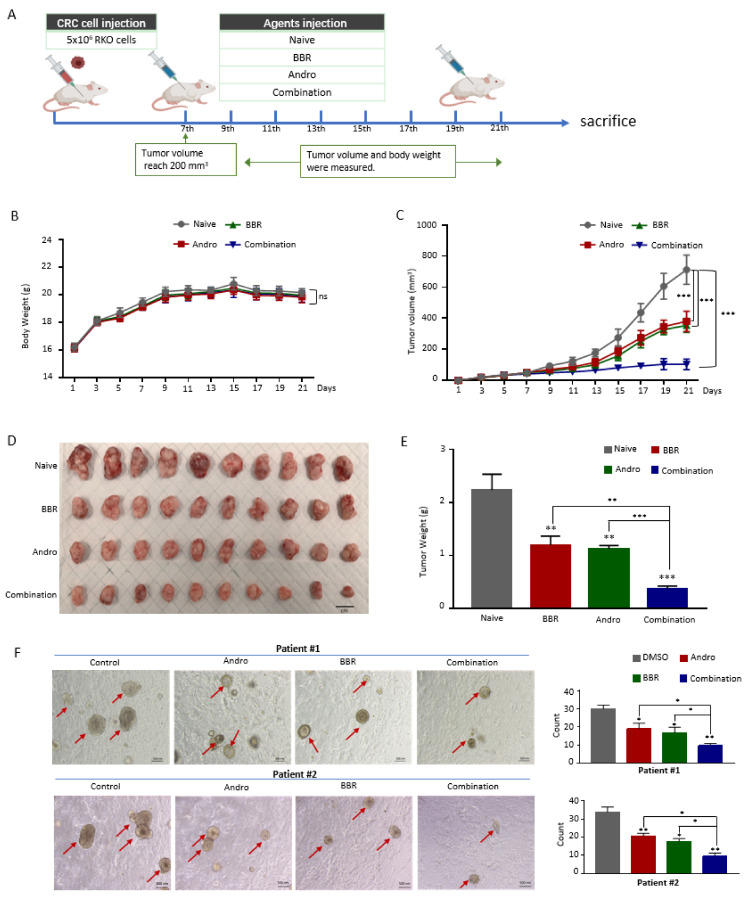
Andrographis enhances the efficacy of berberine in mouse xenografts and patient-derived tumor organoids. (**A**) A schematic diagram of the RKO xenograft model in nude mice and the treatment schedule of berberine, Andrographis, and their combination; (**B**) The bodyweight alterations of the mice in each treatment group were measured at different time-points after inoculation; (**C**) The tumor volume alterations of mice in each treatment group were measured at different time-points after inoculation; (**D**) Representative images of anatomic tumor xenografts dissected from nude mice after they were sacrificed; (**E**) The measurement of the xenograft weights of each treatment group; (**F**) Microscopic images of patient-derived tumor organoids derived from two patients cultured in each treatment group (20× magnification). The organoid count analysis is shown in the right panel. Statistical Significance: * *p* < 0.05, ** *p* < 0.01, *** *p* < 0.001. BBR, berberine; Andro, Andrographis.

The above data demonstrated that berberine and Andrographis in combination enhanced the anti-cancer effect in vitro. To confirm whether Andrographis could enhance the cancer-suppressive properties of berberine in a pre-clinical animal model, we next established a subcutaneous xenograft mouse model as described previously [22]. In brief, RKO-cell-derived xenografts were generated by subcutaneous injection of 5 × 10^−6^ cells. Nude mice bearing RKO-derived xenografts were randomly administered berberine, Andrographis, or a combination every other day. Body weight and tumor volume were measured at the same time until the animals were sacrificed (Figure 3A). No significant weight loss was observed in any of the treatment groups, indicating that the toxicity of the combination medication was acceptable (Figure 3B). The measurements of tumor volumes and anatomic tumor weights showed that, although berberine and Andrographis reduced tumor growth as monotherapy agents, the combination therapy enhanced their anti-cancer effects, suggesting that the cytotoxicity of berberine was enhanced by the addition of Andrographis in the mouse xenografts (*p* < 0.01; Figure 3C–E).

In view of the advantages of the three-dimensional tumor organoid culture model in the research of novel anti-cancer agents, which provide a physiological microenvironment similar to the human body for the cell–cell interaction and response, we next attempted to further confirm our findings using patient-derived tumor organoids. We collected CRC tissues from two patients with CRC, established CRC organoids, and, thereafter, evaluated the enhanced anti-cancer effects of berberine and Andrographis in these organoids. Consistent with our in vitro and in vivo observations, both berberine and Andrographis consistently reduced the number and size of CRC organoids (*p* < 0.05); however, the combination of berberine and Andrographis exhibited a significantly higher degree of anti-growth effects compared to the treatments with individual extracts (*p* < 0.05; Figure 3F), once again confirming our findings from the cell culture and pre-clinical animal model experiments.

### 2.4. The Mutually Enhanced Anticancer Effect of Andrographis and Berberine Is Mediated by Suppressing DNA Replication in Cancer Cells

**Figure 4:**
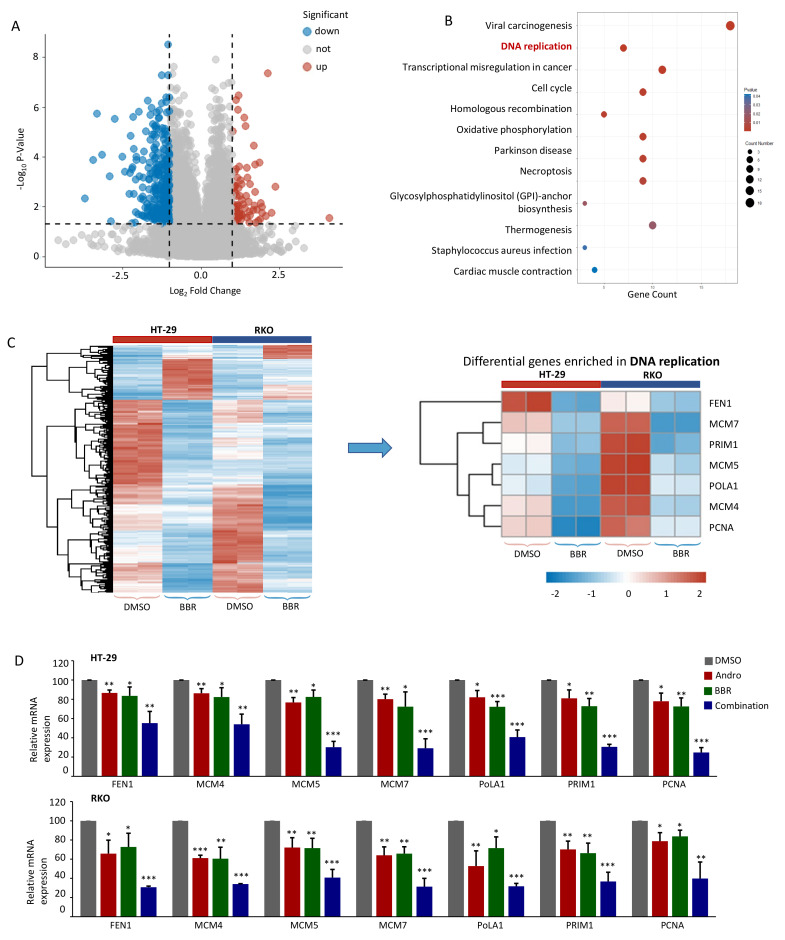
The enhanced anticancer effect of Andrographis and berberine is mediated by the suppression of the DNA replication pathway in CRC. (**A**) The volcano plot obtained from whole-transcriptome profiling analyses of RKO and HT-29 cells after treatment with berberine (|log2 fold change| > 1, *p* < 0.05); (**B**) The bubble diagram of the KEGG pathway analysis based on the significantly dysregulated genes; (**C**) The heatmap of the common differentially expressed genes in both RKO and HT-29 cells and the heatmap of the transcriptional expression of DNA replication-specific genes; (**D**) A bar graph showing the relative mRNA expression of FEN1, MCM7, PRIM1, MCM5, POLA1, MCM4, and PCNA in RKO and HT-29 cells after being treated by berberine, Andrographis and their combination. Statistical Significance: * *p* < 0.05, ** *p* < 0.01, *** *p* < 0.001. BBR, berberine; Andro, Andrographis.

To elucidate the key molecular pathways responsible for the anti-tumorigenic properties of berberine, an RNA-seq was conducted to profile the gene expression alterations in RKO and HT-29 cells following treatment with berberine. The transcriptomic profiling data revealed a large number of differentially expressed genes, including 70 upregulated and 332 downregulated genes in both cell lines following berberine treatment, using a criterion based on fold change and p-values (Figure 4A). A Kyoto Encyclopedia of Genes and Genomes (KEGG) pathway analysis on these significantly dysregulated core targets led us to identify that DNA replication was significantly enriched in both CRC cell lines (Figure 4B). Further analysis of specific genes enriched in DNA replication revealed the significant downregulation of FEN1, MCM7, PRIM1, MCM5, POLA1, MCM4, and PCNA (Figure 4C).

Next, to explore whether the combination therapy with berberine and Andrographis achieved a mutually enhanced anti-CRC effect through their combinatorial regulation of DNA replication, we assessed the expression levels of the differentially expressed genes enriched within the DNA replication pathway in HT-29 and RKO cells after exposure to berberine, Andrographis, and the combination. Interestingly, in RKO and HT-29 cells, berberine and Andrographis simultaneously downregulated the expression of FEN1, MCM7, PRIM1, MCM5, POLA1, MCM4, and PCNA, and the differential gene expression was significantly more suppressed when the two agents were used in combination (*p* < 0.05; Figure 4D), indicating that the enhanced anticancer effect of Andrographis and berberine is mediated by inhibiting DNA replication.

### 2.5. Andrographis and Berberine Cooperatively Inhibit DNA Replication in Mouse Xenografts and Patient-Derived Tumor Organoids

**Figure 5:**
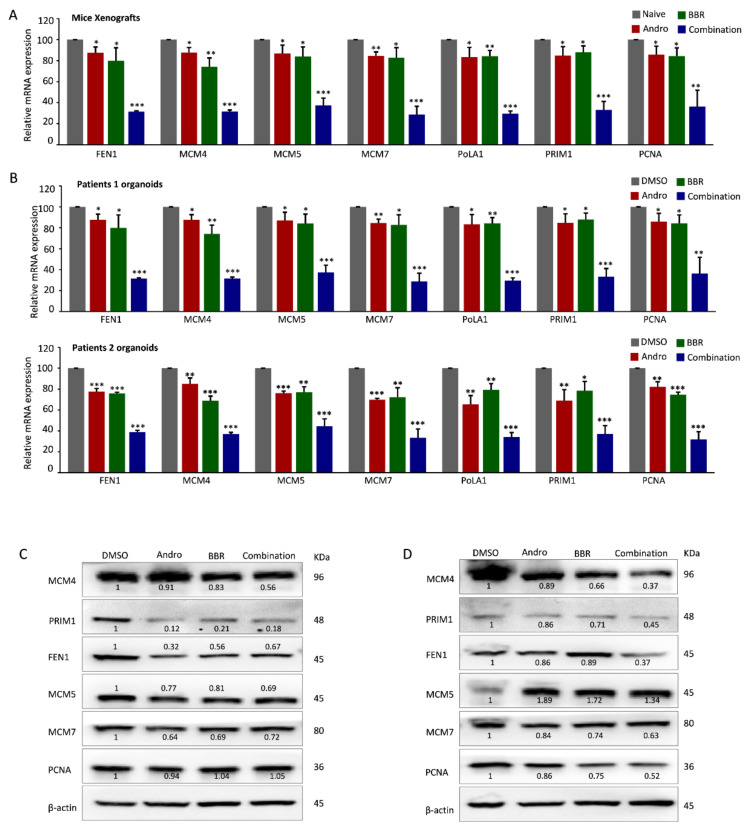
Andrographis and berberine mutually enhance the inhibition of DNA replication in mouse xenografts and patient-derived tumor organoids. (**A**) A bar graph showing the relative mRNA expression of FEN1, MCM7, PRIM1, MCM5, POLA1, MCM4, and PCNA in dissected tumor xenografts after being treated with berberine and/or Andrographis; (**B**) A bar graph showing the relative mRNA expression of DNA-replication-related genes in two strains of patient-derived tumor organoids; (**C**,**D**) The protein immunoblot analysis of DNA-replication-related proteins in RKO cells and dissected tumor xenografts treated with berberine and/or Andrographis. Statistical Significance: * *p* < 0.05, ** *p* < 0.01, *** *p* < 0.001. BBR, berberine; Andro, Andrographis.

Finally, we asked whether the regulations of Andrographis and berberine on DNA replication can also be demonstrated in mouse xenografts and patient-derived tumor organoids. To answer this question, we extracted total RNA from the dissected tumor xenografts and examined the alterations of DNA-replication-related genes in the different treatment groups. In line with our results from the cell lines, the combination treatment with Andrographis and berberine significantly decreased the mRNA expression of FEN1, MCM7, PRIM1, MCM5, POLA1, MCM4, and PCNA vis-à-vis individual treatment (*p* < 0.05; Figure 5A). Furthermore, consistent with our results from the in vitro and in vivo experiments, we were able to successfully confirm the suppression of the expression of key DNA-replication-related genes, even in the tumor organoids, following treatment with Andrographis, berberine, and their combination (*p* < 0.05; Figure 5B).

To determine whether these genes were also suppressed at the post-transcriptional level, A Western blot analysis was performed to evaluate the protein expressions of FEN1, MCM7, PRIM1, MCM5, POLA1, MCM4, and PCNA in RKO cell extracts. Interestingly, among these proteins, we also observed the significantly enhanced downregulation of MCM4 and PRIM1 protein expression in RKO cells treated by the berberine and Andrographis combination (Figure 5C), as well as the same alterations in MCM4 and PRIM1 proteins in tumor xenografts (Figure 5D), highlighting the relevance of DNA replication pathway-associated genes in promoting the anti-cancer efficacy of Andrographis and berberine combination in CRC.

## 3. Discussion

Despite tremendous advances in treatment modalities, CRC remains the third leading cause of cancer-related deaths worldwide [23]. Surgery has been the mainstay of CRC treatment for several decades and is often the sole method to treat the cases of early diagnosis but is no longer effective in the majority of the advanced cases where cancer has already metastasized [7]. In these patients, chemotherapy is the primary treatment to shrink tumors. However, rapidly evolving drug resistance and the severe side-effects of chemotherapeutic agents often reduce the clinical efficacy of various anti-cancer agents [24,25]. Hence, there is always a constant need to develop alternative or synergistic anticancer drugs with minimal side effects and more comprehensive cancer-suppressive effects. The emergence and development of CRC is often a consequence of the acquisition of a vast array of genomic and epigenomic alterations, and not all patients with CRC share similar mutations, making it difficult to design a “one-shoe-fit-all” therapeutic approach. Unlike other types of cancers, CRC can be prevented in the general population through lifestyle interventions and dietary modifications, inspiring both researchers and clinicians to identify all potential therapeutic agents derived from natural sources that could improve survival and outcomes for this malignancy [26]. In the context of cancer treatment, various extracts from natural plant and their derivatives are being extensively studied and proven to be effective as cancer therapeutics [27].

Not surprisingly, of the 175 small molecule anti-tumor agents approved between the 1940s to 2014, almost 50% of them were derived directly from various natural products [28]. As a class of natural isoquinoline alkaloids, berberine has been shown to possess extensive pharmacological activities, such as anti-inflammatory, antioxidant, and anti-diabetes effects [15,29,30]. In addition, berberine has been of interest for years due to its anti-tumor activity of interfering with a variety of characteristics of tumorigenesis and tumor development. Berberine is known to upregulate p53 expression by suppressing the TP53 regulator MDM2 at the post-transcriptional level and induces apoptosis in acute lymphoblastic leukemia cells [31]. Berberine has also been reported to induce apoptosis by increasing reactive oxygen species (ROS) in certain breast cancer cells (MCF-7 and MDA-MBA-231) [32]. Berberine activates mitochondrial apoptosis in hepatocellular carcinoma cells by increasing Bax expression, PT pore formation, and subsequent activation of caspase 3 and 9 signaling pathways. The anti-tumor activity of berberine, either alone or in combination with other chemotherapy regimens, has been demonstrated. Berberine enhances the cytotoxicity and apoptosis of various chemotherapy-induced tumor cells and shows a better therapeutic effect when used in combination with other drugs. For example, berberine could be used as an adjuvant agent with cisplatin to improve the efficacy of inducing necroptosis and apoptosis in ovarian cancer cells. In the current study, we demonstrated that a novel combination of berberine and Andrographis had a mutually reinforcing inhibitory effect on CRC. Furthermore, our data revealed that berberine and Andrographis inhibited intracellular DNA replication by simultaneously affecting key genes, leading to cell cycle arrest, and achieving enhanced inhibitory effects on cell proliferation and colony formation capacity when used in combination.

There are two major ways to inhibit the progression of cancer cell growth: inhibiting cancer cell proliferation and promoting apoptosis and death. Therefore, the agents that inhibit proliferation and/or promote apoptosis of cancer cells are of great therapeutic value. In eukaryotes, DNA replication is a key determinant of chromosome isolation and stability. EdU incorporation is usually used to monitor DNA synthesis during the cell cycle S phase and the situation of DNA replication. In this study, the EdU incorporation assay showed that both berberine and Andrographis decrease DNA replication. This finding was confirmed by the alterations in cell cycle dynamics induced by berberine and Andrographis. The activation of tumor suppressor factors or the loss of oncogenes leads to continuous inhibition of proliferation signals in cancer cells, resulting in the dysregulation of DNA replication and a loss of control over the cell cycle. Therefore, targeting DNA replication has become one of the promising strategies in cancer therapy. Berberine and Andrographis inhibit the process of DNA replication by co-inhibiting the same genes associated with DNA replication, resulting in the inhibition of cell proliferation and colony formation, underscoring the compelling rationale for using these two agents in combination for cancer treatment.

There were several novel aspects of our study. First, while the anti-cancer effects of berberine and Andrographis have been studied in other cancers, limited data exists for their efficacy in colorectal cancer. Second, to the best of our knowledge, no previous studies have performed a genome-wide transcriptomic analysis to perform a systematic delineation of the key growth regulatory pathways that can help us to understand the anti-cancer activities of each preparation. Finally, to the best of our knowledge, there have been no studies that have yet interrogated their combinatorial effects in colorectal cancer, a disease that is intimately linked with dietary and lifestyle factors. One of the potential limitations of our study was that we were unable to demonstrate the specificity of the anti-tumorigenic effects of these preparations in ‘normal’ colon cancer cells. This was primarily due to the fact that most of such cell lines, including the FHC, NCM460, and HCoEpiC cell lines, are not truly normal, as they harbor various genetic and epigenetic alterations due to their neoplastic transformation over the years.

**Figure 6:**
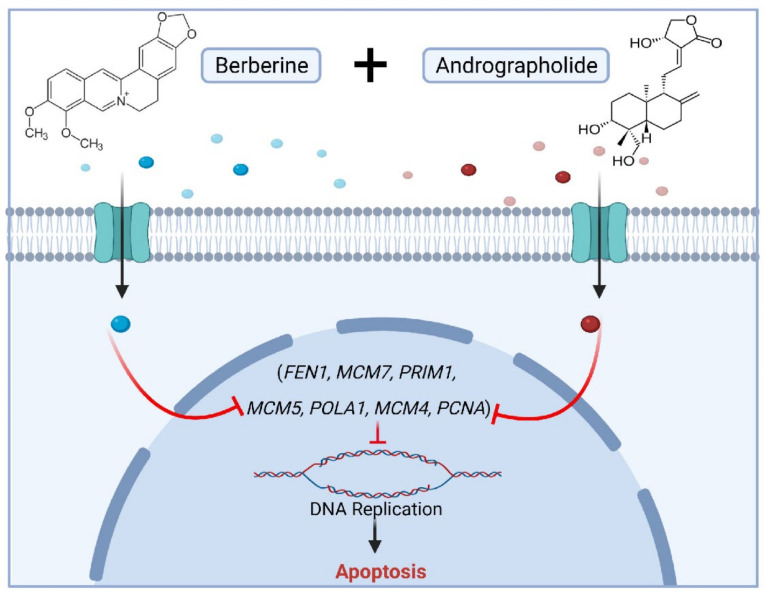
A schematic illustration of the berberine and Andrographis combination treatment showing enhanced anticancer activity by inhibiting DNA replication in CRC cells.

Taken together, our data reveal a potent anti-tumorigenic property of berberine in a series of in-vitro, in-vivo, and patient-derived organoid experimental models and provide multiple layers of evidence supporting the enhanced anticancer effects of berberine combined with Andrographis. For the investigation of the potential mechanism, we profiled the berberine-induced gene expression alterations by RNA-sequencing to confirm that berberine predominantly inhibited DNA replication and to identify several novel target genes targeted by berberine. Furthermore, we found that the mutually enhanced antitumor effect of Andrographis and berberine is achieved through the co-regulation of DNA replication by verifying these genes at the transcriptional and post-transcriptional levels (Figure 6). These findings provide sufficient evidence for targeting DNA-replication-related genes as a promising new therapeutic strategy for CRC. While further analysis is needed in the future, we are excited that the combination of berberine and Andrographis may be a potential therapeutic strategy for patients with CRC in the future.

## 4. Materials and Methods

### 4.1. Cell Lines and Materials

The human CRC cell lines HCT116, SW48, RKO, Caco-2, SW480, and HT-29 were purchased from the American Type Culture Collection (ATCC, Manassas, VA, USA). HCT116, SW480, HT-29, and SW48 were maintained in Dulbecco’s Modified Eagle Medium (DMEM, Gibco, Carlsbad, CA, USA), and Caco-2 and RKO were grown in Roswell Park Memorial Institute 1640 (RPMI-1640, Gibco, Carlsbad, CA, USA). All media contained 10% fetal bovine serum (FBS, Gibco, Carlsbad, CA, USA), supplemented with antibiotics (Penicillin G 100 U/mL, Streptomycin 100 µg/mL) (Sigma–Aldrich, St. Louis, MO, USA) at 37 °C in 5% CO_2_ atmosphere. All cell lines were authenticated by a panel of short tandem repeat markers and known genetic signatures and were periodically tested for mycoplasma.

### 4.2. Herbal Preparations

The two herbal extracts used in this study were *Andrographis paniculata* Burm. F. Nees herb dry native extract (DER 30:1, extraction solvent: 70% ethanol) standardized for the content of andrographolide (20%) and Indian Barberry (*Berberis aristate* DC.) Bark and root extract (DER 40:1, extraction solvent: methanol) standardized for the content of berberine (97%), (Arjuna Natural Pvt. Ltd., Kerala, India). The herbal preparation quality of the extracts was tested by HPLC in accordance with specifications using appropriate reference standards. All analytical methods were validated for selectivity, accuracy, and precision.

Andrographis extracts were dissolved in dimethyl sulfoxide (DMSO, Sigma-Aldrich, St. Louis, MO, USA) to the desired stock concentrations, while berberine extracts were dissolved in double distilled water to stock concentrations. The experimental media were prepared and diluted to appropriate concentrations in complete medium before use, and the final DMSO content did not exceed 0.1%.

### 4.3. Tumor Xenograft Model

Male immunodeficient BALB/c nude mice (five weeks old and weighing 18–20 g) were purchased from Envigo (Houston, TX, USA). To establish the xenograft models, 5 × 10^6^ RKO cells in 100 μL of PBS were subcutaneously inoculated into the left flank of the mouse (*n* = 40) using a 27-gauge needle. When the tumors became visible (about one week after the cell injection), the mice were randomly divided into four groups (n = 10 for each group): berberine (75 mg/kg of body weight), Andrographis (100 mg/kg of body weight), their combination, and a control group (equal amount of PBS) and were given intraperitoneal doses every other day for up to 2 weeks. The tumor size and body weights were measured and calculated every other day. The formula for the tumor volume calculation was V = 0.5 × length × width × width. After the mice were euthanized, each tumor was dissected, weighed, and frozen in liquid nitrogen for subsequent extraction of total RNA and protein. All procedures involving animal experiments were carried out under the National Institutes of Health (NIH) Guide for the Care and Use of Laboratory Animals, 8th edition, and approved by the Institutional Animal Care and Use Committee (IACUC) of City of Hope.

### 4.4. Cell Viability Assay

Cell cytotoxicity in the presence or absence of berberine and Andrographis was assessed by a Cell Counting Kit-8 (CCK-8) assay using a CCK-8 kit (Dojindo, Kumamoto, Japan) as previously described [19]. Briefly, RKO and HT-29 cells were cultured in 100 µL of complete media at a density of 3 × 10^3^ cells/well for 24 h in a 96-well plate. Thereafter, the cells were simultaneously treated in triplicate wells with the appropriate concentration of berberine, Andrographis, their combination, or with the vehicle for untreated controls, for 48 h. The media were replaced with CCK-8 solution at the indicated time points and incubated for 2 h. The absorbance was measured at 450 nm (OD450) using a microplate reader (Molecular Devices, San Jose, CA, USA). The inhibition rates of cell viability relative to vehicle controls were calculated using the following formula:

$$\mathrm{Cell}\;\mathrm{inhibition}\;\left(\%\right)=\frac{\left(\mathrm{control}\;\mathrm{OD}\;\mathrm{value}\right)-\left(\mathrm{Experimental}\;\mathrm{OD}\;\mathrm{value}\right)}{\mathrm{control}\;\mathrm{OD}\;\mathrm{value}}\times 100$$

The sigmoidal dose–response models were fitted by GraphPad Prism statistical software (GraphPad Software Inc., San Diego, CA, USA), and the IC_50_ value and slope (m) of the concentration–response curves were calculated. The CI method described by Chou and Talalay was used to evaluate the synergy between berberine and Andrographis by CompuSyn software (ComboSyn, Inc., Paramus, NJ, USA).

### 4.5. Cell Cycle Analysis

DNA staining was performed using the Muse™ Cell Cycle Kit (Merck Millipore, Guyancourt, France) to determine the percentages at each stage of the cell cycle (G0/G1, S, and G2/M). Briefly, RKO and HT-29 cells were plated in 6-well plates at a density of 5 × 10^4^ cells in 2 mL of complete media and cultured for 24 h, then were treated with the appropriate concentration of berberine, Andrographis, their combination, or vehicle controls for 48 h. Thereafter, cells were collected using 0.1% trypsin, rinsed twice with ice-cold PBS, and then fixed with 200 µL of ice-cold 70% ethanol and incubated at −20 °C for 3 h. After two washes with ice-cold PBS, cells were stained with 200 µL of Muse™ cell cycle reagent in room temperature darkness for 30 min. The cell cycle analysis results were obtained using the Muse™ Cell Analyzer (Merck Millipore, Guyancourt, France).

### 4.6. Colony Formation Assay

RKO and HT-29 cells were seeded into 6-well plates at a low density (800 cells/well) and maintained in complete medium for one week. Thereafter, these were treated with the appropriate concentrations of berberine, Andrographis, or vehicle controls for another week. Fresh cell culture medium was replaced every 3 days for continuous culture. Then, the colonies were immobilized with methanol for 10 min and stained with 0.1% crystal violet (ACROS Organics, Gujarat, India). The numbers of staining colonies were counted under a microscope. The experiments were performed in triplicate.

### 4.7. RNA Extraction and Real-Time qPCR

Total RNA was extracted with TRIzol reagent (Invitrogen, Carlsbad, CA, USA) from CRC cells, chopped xenograft fragments (approx. 1 mm^3^ in size), and dissociated organoids derived from patients. After testing the purity and concentration using a Nanodrop 2000 spectrophotometer (NanoDrop Technologies, Inc., Wilmington, DE), total RNA was reverse transcribed into cDNA by a High-Capacity cDNA Reverse Transcription Kit (Applied Biosystems, Foster City, CA, USA). Quantitative PCR (qPCR) analyses were performed using SensiFAS SYBR Lo-ROX Kit (Bioline, London, UK) on a CFX96 real-time PCR detection system. For target gene amplification analysis, the relative expressions of the target genes were normalized against the housekeeping gene β-actin via the published comparative 2^−∆∆Ct^ method. The specific sequences of the target gene primers are presented in Table 1.

### 4.8. Western Blot Analysis

Protein samples were obtained from CRC cells, chopped xenograft fragments (approx. 1 mm^3^ in size), and dissociated organoids derived from patients using RIPA Lysis and Extraction Buffer (ThermoFisher Scientific, Waltham, MA, USA) containing a protease inhibitor cocktail (ThermoFisher Scientific, Waltham, MA, USA) and incubated on ice for 30 min. Laemmli’s buffer (Bio-Rad, Hercules, CA, USA) containing mercaptoethanol (Bio-RAD, Hercules, CA, USA) was then added to the protein samples and heated and boiled for 10 min. Proteins were separated by electrophoresis in a 10% sodium dodecyl sulfate-polyacrylamide gel, then electro-transferred onto a 0.45 µm polyvinylidene fluoride (PVDF) membrane (Cytiva, Marlborough, MA, USA), followed by being blocked in 5% bovine serum albumin (BSA, Sigma-Aldrich, St. Louis, MO, USA) blocking solution for 1 h. PVDF membranes were thereafter incubated overnight at 4 °C with diluted primary antibodies, which are listed as follows: anti-β-actin (#A2228, Sigma-Aldrich, St. Louis, MO, USA), anti-PCNA (#SC-7907, Santa Cruz, Dallas, TX, USA), anti-FEN1 (#82354S, Cell Signaling Technology, Danvers, MA, USA), anti-PRIM1 (#4725, Cell Signaling Technology, Danvers, MA, USA), anti-MCM4 (#ab124836, Abcam, Cambridge, UK), anti-MCM5 (#ab17967, Abcam, Cambridge, UK), and anti-MCM7 (#ab52489, Abcam, Cambridge, UK). After washing with 1% Tween-20 (Sigma-Aldrich, St. Louis, MO, USA) PBS three times, the membranes were incubated with horseradish peroxidase (HRP)-conjugated secondary antibodies (#7074 or #7076, Cell Signaling Technology, Danvers, MA, USA) at room temperature for 1 h. Immunoblots were visualized using an HRP-based chemiluminescence kit (ThermoFisher Scientific, Waltham, MA, USA) by Gel Imaging Systems (Bio-Rad, Hercules, CA, USA). The band intensity was quantified by Image J software and expressed as the ratio of the β-actin band intensity.

### 4.9. Patient-Derived Organoid Cultures

Two strains of human organoids derived from patients with CRC were prepared, isolated, cultured, and passaged as described previously [22]. In accordance with the Declaration of Helsinki, all patients provided written informed consent, and all procedures involving patient-derived organoids were approved by the institutional review board. For treatments, the organoids were randomly divided into 4 groups, and cultured with IntestiCult Organoid Growth Medium (STEMCELL Technologies, Vancouver, BC, Canada) supplemented by corresponding concentrations of berberine, Andrographis, their combination, or vehicle controls for 10 days. Subsequently, the treated organoids were observed and counted under a microscope and harvested by Gentle Cell Dissociation Reagent (STEMCELL Technologies, Vancouver, BC, Canada). The harvested organoids were aliquoted and frozen in liquid nitrogen for subsequent total RNA and protein extraction.

### 4.10. RNA-Sequencing

RNA-sequencing (RNA-seq) was performed following the same method as previously reported [19]. In brief, total RNAs were extracted from RKO cells (2 × 10^6^) that were treated for 48 h with berberine (2 #mM), Andrographis (15 μg/mL), their combination, or vehicle controls using an miRNeasy kit (Qiagen, Hilden, Germany) according to the manufacturer’s instructions. RNA-seq libraries were prepared after RNA concentration and quality were evaluated by a Bioanalyzer 2100 Expert (Agilent Technologies, Santa Clara, CA, USA). Next, 1μg of qualified RNA from each sample was used as the input material to generate ribosomal RNA-depleted libraries via the TruSeq RNA Sample Prep Kit (Illumina, San Diego, CA, USA). The purified library products were then assessed by a Bioanalyzer DNA High Sensitivity Kit (Agilent Technologies, Santa Clara, CA, USA) to determine its size distribution and concentration and verified by paired sequencing (150 bp at each end). The analysis was performed in the HiSeq X-TEN system (Illumina, San Diego, CA, USA).

The RNA-seq data were analyzed using R (version 4.0.2) software package edgeR for normalization and differential expression analysis. Clean reads were obtained by removing those containing adapters and low-quality reads and they were then independently mapped to the reference genome. The read counts of the genes were first standardized by fragments per kilobase of transcript per million mapped reads (FPKM). Significant differentially expressed genes (DEGs) were selected by a threshold of false discovery rate (FDR) < 0.05 and absolute fold-change > 2.0.

### 4.11. Statistical Analysis

Data are presented as descriptive statistics in tables and graphs and showed by the means ± standard deviations (SD) of three independent experiments. GraphPad Prism 8.0 (GraphPad Software, Inc., La Jolla, CA, USA) was used for statistical analysis, and the Sigmoidal dose–response model was fitted to calculate the IC_50_ values. CompuSyn software (CompuSyn Inc., Paramus, NJ, USA) was applied to calculate the Chou–Talalay CI. Comparisons between groups were performed by Student *t*-test, and comparison of effects in two (or more) groups over the multiple time points were performed by a two-way ANOVA. A *p*-value < 0.05 was considered statistically significant between experimental groups.

## Figures and Tables

**Table 1:** Primers for qPCR analysis.

Gene Name	Primer	Sequence
β-actin	Forward	5′-CACCATTGGCAATGAGCGGTTC-3
	Reverse	5′-AGGTCTTTGCGGATGTCCACGT-3
FEN1	Forward	5′-ACTAAGCGGCTGGTGAAGGTCA-3
	Reverse	5′-GCAGCATAGACTTTGCCAGCCT-3
MCM4	Forward	5′-CTTGCTTCAGCCTTGGCTCCAA-3
	Reverse	5′-GTCGCCACACAGCAAGATGTTG-3
MCM5	Forward	5′-GACTTACTCGCCGAGGAGACAT-3
	Reverse	5′-TGCTGCCTTTCCCAGACGTGTA-3
MCM7	Forward	5′-GCCAAGTCTCAGCTCCTGTCAT-3
	Reverse	5′-CCTCTAAGGTCAGTTCTCCACTC-3
PoLA1	Forward	5′-GGACCAACACATCTAGCCTGGA-3
	Reverse	5′-GGTCTGGTTTCAAAGCCATTGCC-3
PRIM1	Forward	5′-TATCGCTGGCTCAACTACGGTG-3
	Reverse	5′-CACTCTGGTTGTTGAAGGATTGG-3
PCNA	Forward	5′-CAAGTAATGTCGATAAAGAGGAGG-3
	Reverse	5′-GTGTCACCGTTGAAGAGAGTGG-3

## Data Availability

Data is contained within the article.
